# 2656. Longitudinal Trends in Attributable Causes of Death Among US Veteran Patients with Documented SARS-CoV-2 Infection between January 2021 and March 2023

**DOI:** 10.1093/ofid/ofad500.2267

**Published:** 2023-11-27

**Authors:** Caitlin A Trottier, Jennifer La, Lucy Li, Nathanael Fillmore, Paul Monach, Westyn Branch-Elliman, Shira Doron

**Affiliations:** Tufts Medical Center, Boston, Massachusetts; VA Boston Healthcare System, Boston, Massachusetts; Beth Israel Deaconess Medical Center, Brookline, Massachusetts; VA Boston Healthcare System, Boston, Massachusetts; VA Boston Healthcare System, Boston, Massachusetts; VA Boston Healthcare System, Boston, Massachusetts; Tufts Medical Center, Boston, Massachusetts

## Abstract

**Background:**

COVID-19 was the third leading cause of death in the US in 2020, and as of April 2023, >1,000,000 deaths have been attributed to this disease. With widespread immunity and medical countermeasures, the attribution of a death to COVID-19 has become more complex. The aim of this study was to examine the trends in the proportion of deaths attributed to COVD-19 in both vaccinated and unvaccinated patients following a documented positive SARS-CoV-2 test.

**Methods:**

We used an electronic tool, developed using a chart reviewed sample of national Veterans Affairs (VA) data and validated on manually adjudicated cases at Tufts Medical Center, to classify deaths as attributable to COVID-19; both deaths in which COVID-19 contributed and was the primary cause of death were captured by the tool. The tool comprises a 3-point index (remdesivir, dexamethasone, baricitinib, or tocilizumab receipt; hypoxemia with SpO2 < 90% or supplementary oxygen > 2L), for which a score 2-3 has a high positive predictive value (PPV, 0.82-0.95) for COVID-19-related death. We then applied the tool to classify all known deaths among VA patients within 30 days of a positive SARS-CoV-2 test between January 2021 and March 2023 to measure longitudinal trends.

**Results:**

A decrease in the proportion of deaths attributable to COVID-19 between August 2021 and March 2023 was identified (p-value for trend, < 0.001). Despite an overall higher number of deaths attributable to COVID-19 in the vaccinated population (6081 versus 4697, and 2650 versus 488 in the 12 months starting March 2022, Figure 1), the proportions of deaths attributable to COVID-19 (Figure 2) was similar in both cohorts, trending from > 70% to < 50%.

**Figure:**
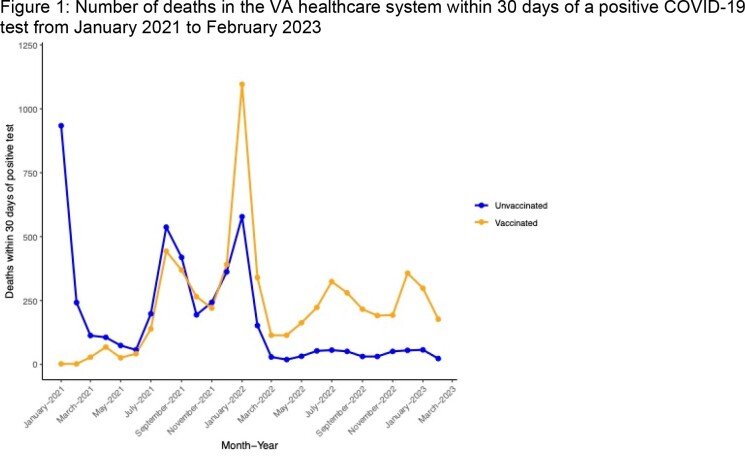

**Figure:**
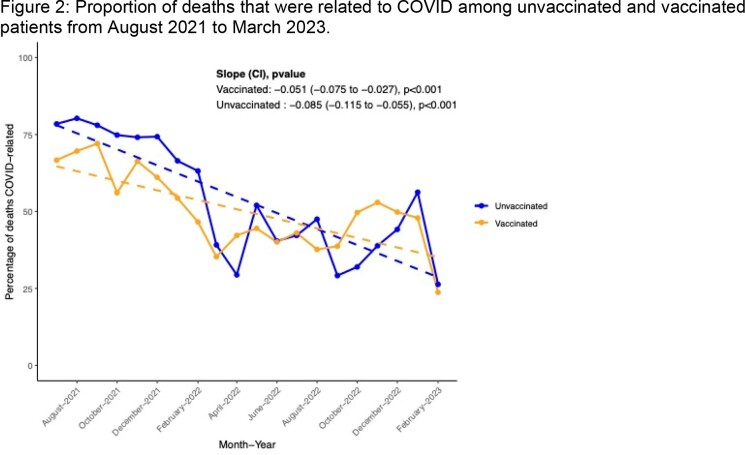

**Conclusion:**

Throughout the pandemic, there have been a myriad of measures used to guide the ongoing public health response including case rates, hospitalization rates, and death rates. Because chart review is the only way to more definitively determine whether a hospitalization or death is directly or indirectly caused by COVID-19, much of the data, obtained through more crude means, lack accuracy. Our electronic tool gives a closer approximation to what is obtained by chart review than the other commonly used measures and reveals that overall, the proportion of attributable deaths has universally decreased.

**Disclosures:**

**Paul Monach, MD, PhD**, HI-Bio: Advisor/Consultant **Westyn Branch-Elliman, MD, MMSc**, Gilead Sciences: Grant/Research Support

